# Evolving disparities in exercise-related physical activity among Chinese children and adolescents: evidence from a national repeated cross-sectional analysis, 2014–2022

**DOI:** 10.3389/fpubh.2026.1883019

**Published:** 2026-10-14

**Authors:** Ming Ming Guo, Binping Gong, Rui Li, Zhen Wang, Honghan Xu, Di Zhang

**Affiliations:** 1 Department of Physical Education, College of Education of Future, Beijing Normal University, Zhuhai, China; 2 Guangdong Vocational Academy of Art, School of Culture Tourism, Guangzhou, China

**Keywords:** children and adolescents, China, disparities, physical activity, trends

## Abstract

**Background:**

Global studies show low levels of physical activity (PA) among children and adolescents, yet evidence on long-term national trends and subgroup disparities in China is limited. This study examined exercise-related PA trends among Chinese children and adolescents from 2014 to 2022 and explored temporal changes in subgroup disparities.

**Methods:**

A national repeated cross-sectional analysis was conducted using data from five waves (2014–2022) of the China Family Panel Studies (CFPS), including 16,223 observations from 7,982 children and adolescents aged 10–18 years. Exercise-related PA time was assessed using self-reported frequency and duration; ≥60 min/day was an exploratory threshold, not WHO guideline adherence. Survey-weighted descriptive statistics, categorical-wave comparisons, interaction tests, and age-adjusted logistic regression models examined overall trends and disparities by school level, gender, income, urban–rural residence, and region.

**Results:**

Between 2014 and 2018, unadjusted weighted average daily PA time among Chinese children and adolescents increased by 3.0 min/day (95% CI, 0.1, 6.0), although this small difference was not robust in sensitivity analyses. From 2020 to 2022, PA increased by 10.2 min/day (95% CI, 7.5, 12.9). Gender-based differences in PA remained consistently significant throughout, with boys being more active than girls. Gender differences varied by wave; regional variation was less consistent in sensitivity analyses. The primary analysis did not support changing disparities by school level, urban–rural residence, or income. Males, children from higher-income families, and urban residents had higher adjusted odds of reporting ≥60 min/day of exercise-related PA.

**Conclusion:**

Exercise-related PA among Chinese children and adolescents increased between 2020 and 2022. The questionnaire change in 2020 limits interpretation of the 2018–2020 difference. Targeted policies should continue to promote PA, especially for children from low-income families. Efforts should also consider demographic-specific strategies to address persistent inequalities in PA participation.

## 1 Introduction

Although the importance of physical activity (PA) for the physical and mental health of children and adolescents is widely recognized, PA levels remain low in many settings (1, 2). For example, Guthold et al. analyzed surveys from 2001 to 2016 and estimated that 81.0% (95% uncertainty interval, 77.8–87.7) of school-attending adolescents aged 11–17 years worldwide were insufficiently active in 2016; the corresponding estimate for China was 84.3% (81.1–87.0) (1). Globally, insufficient activity decreased among boys but did not change significantly among girls over this period (1). Neville et al. further found a 20% drop in global PA levels during the COVID-19 pandemic compared to pre-pandemic levels (3).

Previous studies have shown that factors such as age, gender, urban–rural residence, geographic region, socioeconomic status (SES), and environmental and policy contexts are associated with PA among children and adolescents. For instance, older adolescents often engage in less PA alongside factors such as reduced physical education (PE) classes, increased academic pressure, and longer sedentary time (4); boys are generally more active than girls, with sociocultural factors potentially contributing to these differences (1, 5); rural children and adolescents have lower PA participation than urban peers in Chinese survey evidence (6); and weather conditions and SES are associated with PA levels (7, 8). However, further evidence on how these subgroup disparities in PA have changed over time is needed to identify emerging high-risk groups and to inform evaluations of whether policies and school-based initiatives are narrowing—or widening—inequalities.

National evidence from China includes the 2016 Physical Activity and Fitness in China—The Youth Study (6) and a study of PA trends from 2017 to 2019 (9). Evidence during the COVID-19 era is mixed: a Shanghai study reported lower PA between January and March 2020 (10), whereas annual school surveys reported increases during 2019–2021 followed by a decline in 2022 (11). These studies differ in populations, timing and measurement. Changes in transport, sedentary leisure, and access to safe, affordable PA opportunities may link socioeconomic transformation with PA (8). This context of rapid national development offers an opportunity to examine how children’s and adolescents’ PA patterns relate to large-scale social change. The present study extends this evidence by examining five CFPS waves from 2014 to 2022 with household socioeconomic information.

To address the research gap on evolving PA disparities among children and adolescent subgroups, this study examined wave-specific patterns in exercise-related PA among Chinese children and adolescents from 2014 to 2022, focusing on overall changes, subgroup differences, and their temporal patterns using nationally representative data. Importantly, beyond extending the literature, these results can provide actionable guidance for practice and policy in at least three ways: (1) identifying which subgroups (e.g., by school level, gender, SES, urban–rural residence, and region) should be prioritized for targeted school- and community-based interventions; (2) informing more equitable allocation of PA-promoting resources (e.g., facilities, after-school programs, and teacher support) toward groups and areas where disparities persist or widen; and (3) offering population-level benchmarks that can be used to monitor progress and inform future evaluations of policy initiatives and major disruptions (e.g., the COVID-19 era) in relation to youth PA. Based on prior evidence, it was hypothesized that exercise-related PA would increase before the COVID-19 pandemic but decline during the pandemic era, and that disparities by gender and socioeconomic status would persist over time. Because the questionnaire changed in 2020, the wave comparisons do not isolate pandemic effects.

## 2 Materials and methods

### 2.1 Data source and sample composition

This study employed a repeated cross-sectional design using data from the China Family Panel Studies (CFPS), a nationally representative longitudinal household panel survey administered by the Institute of Social Science Survey at Peking University (12). The CFPS sample was drawn from 25 provincial-level regions across China, collectively covering approximately 94.5% of the national population (12, 13). It collects information at individual, family, and community levels on education, employment, income and expenditure, health, family relationships, and living conditions (12).

The CFPS gathers detailed information through computer-assisted personal interviews, and implements rigorous post-survey quality control procedures—including telephone validation, audio verification, and statistical consistency checks—to ensure the reliability of the data (12, 13). Interviews are conducted by trained interviewers using face-to-face or telephone modes; the latter predominated in the 2020 and 2022 analytic waves. The child self-response module uses self-reported answers. Data collection occurs biennially, with seven waves conducted between 2010 and 2022. The CFPS protocol received ethical approval from the Peking University Biomedical Ethics Review Committee (IRB00001052-14010) (14).

This study utilized data from children and adolescents aged 10–18 who participated in the 2014, 2016, 2018, 2020, and 2022 waves of the CFPS (18,106 person-wave observations). We did not include the 2010 and 2012 waves because PA was measured only by exercise frequency in those waves, without information on exercise duration. As our primary outcome required estimating exercise-related PA time (minutes/day), the absence of duration data in 2010 and 2012 precluded harmonization with the later waves and would not allow valid comparisons or trend analyses across 2014–2022. We excluded 1,485 observations without interpretable PA information and a further 398 without a positive national cross-sectional weight. The descriptive sample comprised 16,223 observations from 7,982 individuals (7,641 female observations), with a mean age of 13.84 years (SD = 2.58). Of these individuals, 4,657 contributed to more than one wave; no duplicate person-wave records were found. Covariate-adjusted models used 14,281 observations from 7,226 individuals with complete covariates. We further compared participants included in the final analysis with those excluded due to missing data. Differences were observed across demographic characteristics, particularly age and school-level availability, so exclusion may introduce selection bias rather than remove it. Missing values were not imputed. This limitation was acknowledged and discussed (see Supplementary Table S1 and Limitations).

### 2.2 Assessment of physical activity

PA was assessed using two self-reported items from the CFPS questionnaires, which primarily capture exercise/fitness-related PA (including PE classes for students), rather than overall PA across all activity domains.

For the 2014–2018 waves, participants were asked: (1) “Including PE classes, how many times did you exercise in the past week?” and (2) “Including PE classes, how many total hours did you exercise in the past week?” Interviewer instructions for these waves provided examples of the targeted activities (e.g., running/jogging, ball games, swimming, walking, tai chi, and other exercise activities), indicating that the items were intended to reflect exercise-type PA.

For the 2020–2022 waves, the PA module was revised to: (1) “Excluding cycling or walking solely for commuting to work, but including PE classes, how often did you participate in physical fitness–related recreational PA in the past 12 months (usual pattern)?” Response options ranged from “less than once per month” to “twice or more per day.” (2) “In the past 12 months, on average, how many minutes did you exercise each time?” According to the CFPS codebook and interviewer notes, these items refer to PA undertaken for the purpose of strengthening the body or promoting mental well-being, and explicitly exclude walking or cycling performed solely for commuting to work.

In this study, PA was operationalized as exercise-related PA, without assuming a consistent intensity. For 2014–2018, weekly PA duration was derived directly from the reported total exercise hours in the past week and converted to minutes. For 2020–2022, weekly PA minutes were estimated by converting the categorical frequency responses into sessions per week and multiplying by the reported typical minutes per session. Frequency categories were mapped to weekly sessions as follows: “less than once per month” = 0.125/week; “at least once per month but less than once per week” = 0.5/week; “1–2 times per week” = 1.5/week; “3–4 times per week” = 3.5/week; “≥5 times per week” = 5.5/week; “once per day” = 7/week; “twice or more per day” = 14/week; and “never” = 0/week. Weekly PA minutes were then divided by seven to obtain average daily PA time. The secondary binary outcome classified participants as reporting ≥60 or <60 min/day of exercise-related PA. This exploratory duration threshold does not indicate WHO guideline adherence or physical inactivity. WHO recommends an average of ≥60 min/day of mostly aerobic MVPA for ages 5–17, with vigorous and muscle- and bone-strengthening activities on at least 3 days/week; adult recommendations apply from age 18 (2). The CFPS items do not consistently measure intensity or these strengthening components. Explicit reports of no exercise were coded as zero even when duration was structurally skipped; other nonresponse remained missing. The descriptive sample included 5,269 explicit zero-exercise observations. Assigned frequency scores are approximations, including the open-ended highest category; alternative scores are reported in Supplementary Table S2.

Notably, the CFPS PA items were revised in 2020, resulting in differences in reference period and response format between the 2014–2018 and 2020–2022 waves. We therefore interpret changes that span the 2018–2020 transition with caution and discuss the marked difference between 2018 and 2020 as potentially reflecting both real-world change and methodological differences. Converting both item sets to minutes/day does not establish equivalence, so comparisons within 2014–2018 and within 2020–2022 are emphasized. Without a calibration sample answering both versions for the same reference period, measurement and behavioral changes cannot be separated.

Previous studies have demonstrated the feasibility of using these CFPS PA items to estimate PA in large-scale population surveys, and multiple peer-reviewed CFPS-based studies have applied them in analyses of Chinese children and adolescents and other populations (15–19). Although direct psychometric validation evidence for these items is currently unavailable, previous use establishes feasibility rather than psychometric validity or equivalence across questionnaire versions.

### 2.3 Covariates

This study included several covariates: survey year, age, school level, gender, household income, urban–rural residence, and geographic region. Individual cross-sectional weights were used for survey estimation, not entered as covariates. Information on survey year, age, school level, gender, and residence type was directly obtained from the CFPS questionnaires.

Specifically, school level was categorized into primary, middle (lower secondary), and high school (upper secondary, including vocational and technical programs). These groups reflect school environments and approximate ages 10–12, 13–15, and 16–18, respectively, but are not direct definitions of childhood and adolescence. Age was additionally adjusted continuously; age-band descriptions and an age 10–17 sensitivity analysis are reported in Supplementary Tables S3, S5. Records outside these school stages or with an unavailable stage were retained in overall descriptions but excluded from covariate-adjusted models. The urban–rural classification in CFPS follows the official statistical standards of the National Bureau of Statistics of China. This classification is primarily based on settlement patterns and the built environment (i.e., whether an area forms a continuous built-up zone with urban infrastructure). Administrative divisions provide the geographic units for classification, with specified exceptions for some separate settlements based on population size and land use.

Household income was obtained by linking children and adolescents to family-level data reported by parents or guardians. Household income was then categorized within each survey wave based on the unweighted distribution of total reported household income among age-eligible youth records with observed income: values at or above the 70th percentile were classified as high-income, values at or below the 30th percentile as low-income, and values between these cutpoints as middle-income. Special negative codes and other missing income values were treated as missing. These are sample-relative youth-record groups, not weighted national household quantiles or income adjusted for household size; ties and weighting can produce proportions different from 30/40/30.

In addition, geographic region was divided into seven categories—North, Northeast, East, Central, South, Southwest, and Northwest—based on administrative divisions and provincial locations, reflecting variations in natural, social, and economic conditions (20). The complete province mapping is provided with the analysis code.

These demographic variables were included as covariates to account for their potential confounding effects on PA outcomes. Moreover, year-by-subgroup interaction analyses were conducted separately by school level, gender, income, urban–rural residence, and region, thereby also allowing us to examine their potential role as effect modifiers of temporal PA trends.

Finally, individual cross-sectional weights were constructed by the CFPS team using survey-specific sampling and nonresponse adjustments; calibration procedures and scaling differ across releases (12, 13). The recommended post-stratified national weight was used for 2020 (21). Before pooling, positive weights were rescaled to the same total within each age-eligible wave, avoiding arbitrary differences in file scale; wave-specific means and proportions are unchanged by this scaling. Original 2020 nonresponse-adjusted weights were examined in a sensitivity analysis. Weight variables are listed in Supplementary Table S2.

### 2.4 Statistical analysis

Descriptive statistics were applied to summarize sample distributions across survey waves. Weighted percentages were derived using individual cross-sectional weights from the CFPS to estimate national subgroup proportions, along with weighted average daily PA time and corresponding 95% confidence intervals (CIs) for each survey year. The continuous outcome described exercise duration, while weighted percentages reporting ≥60 min/day summarized an exploratory exercise-specific threshold. Descriptive analyses used all observations with interpretable PA and positive weights; mutually adjusted models used complete covariates. Unadjusted wave contrasts were calculated as differences between survey-weighted means.

Temporal trends were evaluated using survey-weighted linear regression with survey year coded as a categorical variable (2014, 2016, 2018, 2020, 2022), without assuming linear change per calendar year. The dependent variable was daily PA minutes. We emphasized differences within the 2014–2018 and 2020–2022 questionnaire periods, rather than imposing a 2018 pandemic breakpoint. Models included survey year, continuous age, school level, gender, household income, urban–rural residence, and geographic region. To further examine subgroup-specific patterns, separate models added year × school level, gender, household income, urban–rural residence, or region interactions. Joint Wald F tests assessed whether each set of subgroup differences varied by wave; Holm adjustment covered the five omnibus tests. Within-wave adjusted differences and pointwise 95% CIs were also reported.

Additionally, survey-weighted logistic regression models were used on pooled data from 2014 to 2022 to assess associations between reporting ≥60 vs. <60 min/day of exercise-related PA and key demographic factors. These regression models simultaneously included categorical survey year, continuous age, school level, gender, household income, urban–rural residence, and geographic region to control for potential confounding. The reported odds ratios (ORs) and 95% CIs are mutually adjusted associations, not causal effects or risk ratios. These results are reported separately from subgroup mean-difference comparisons.

Sampling uncertainty was estimated by with-replacement Taylor linearization, aggregating weighted estimating-function contributions within stable baseline primary sampling units (PSUs) and centering PSU totals within six independent sampling-frame groups. All 18,106 eligible observations linked to 176 PSUs. The full PSU grid was retained for domain analyses; using a common PSU across waves allowed for within-PSU dependence, including repeated individuals. Mean and model CIs used 170 design degrees of freedom, while proportion CIs used a logit transformation. Joint tests used ordinary Wald F statistics. The public identifiers did not reconstruct finer implicit strata or finite-population corrections.

Sensitivity analyses restricted age to 10–17, excluded reports above 180 min/day, varied later-wave frequency scores and the 2020 weight, omitted age adjustment, additionally adjusted for interview mode, and compared subgroup interactions separately within the two questionnaire periods (Supplementary Table S5). These checks assess selected analysis assumptions; interview-mode adjustment cannot identify the questionnaire-change effect.

All analyses were performed using Python (version 3.12) with NumPy, pandas, and SciPy (22). A two-tailed *p*-value < 0.05 was considered statistically significant, using Holm-adjusted *p*-values for the five primary interaction tests. Reproducible code, variable mappings, aggregate results, and software versions are supplied.

## 3 Results

### 3.1 Participant characteristics

Table 1 presents the demographic characteristics of the 16,223 observations included in the descriptive sample, with annual sample sizes ranging from 2,703 to 3,730. The table also details the unweighted counts and weighted percentages of participants across different survey years by school level, gender, household income, urban–rural residence, and geographic region.

**Table 1:** Sample size and weighted percentages of exercise-related physical activity data among Chinese children and adolescents, 2014–2022^a^^,^^b^.

	2014	2016	2018	2020	2022
Overall	3,730	3,569	3,444	2,703	2,777
School level					
Primary	1,376 (28.6%)	1,461 (26.5%)	1,408 (37.8%)	1,081 (45.1%)	1,120 (41.6%)
Middle	1,233 (29.9%)	1,146 (29.2%)	1,134 (33.0%)	931 (28.8%)	847 (30.5%)
High	713 (28.0%)	684 (34.4%)	664 (20.4%)	560 (20.5%)	692 (23.1%)
Other / unavailable	408 (13.5%)	278 (9.9%)	238 (8.8%)	131 (5.7%)	118 (4.9%)
Gender					
Female	1,786 (46.5%)	1,662 (51.6%)	1,616 (47.6%)	1,288 (48.4%)	1,289 (46.3%)
Male	1,944 (53.5%)	1,907 (48.4%)	1,828 (52.4%)	1,415 (51.6%)	1,488 (53.7%)
Income					
Low	1,100 (27.8%)	1,102 (25.0%)	1,183 (31.3%)	767 (25.5%)	775 (25.5%)
Middle	1,131 (28.6%)	1,328 (37.2%)	1,153 (33.6%)	937 (33.3%)	912 (31.3%)
High	1,370 (39.9%)	1,074 (36.3%)	995 (31.7%)	803 (34.3%)	902 (35.7%)
Other / unavailable	129 (3.8%)	65 (1.5%)	113 (3.4%)	196 (6.9%)	188 (7.5%)
Area					
Rural	2,111 (42.2%)	2,034 (24.5%)	1,972 (47.1%)	1,508 (38.3%)	1,540 (47.0%)
Urban	1,561 (55.6%)	1,508 (74.6%)	1,447 (51.9%)	1,127 (57.9%)	1,226 (52.6%)
Other / unavailable	58 (2.3%)	27 (0.9%)	25 (0.9%)	68 (3.7%)	11 (0.4%)
Region					
North	399 (16.7%)	392 (15.7%)	377 (16.4%)	338 (16.7%)	391 (19.7%)
Northeast	393 (8.7%)	349 (10.2%)	295 (8.6%)	229 (8.8%)	198 (6.3%)
East	540 (19.3%)	528 (19.2%)	526 (18.7%)	422 (19.3%)	436 (19.5%)
Central	680 (14.3%)	669 (16.4%)	637 (16.0%)	486 (16.2%)	563 (15.3%)
South	550 (13.5%)	437 (12.6%)	446 (12.2%)	378 (14.1%)	350 (13.7%)
Southwest	550 (22.8%)	577 (21.4%)	568 (23.5%)	395 (20.2%)	379 (20.9%)
Northwest	618 (4.6%)	617 (4.6%)	595 (4.6%)	455 (4.8%)	460 (4.5%)

a Participant characteristics are presented by survey wave as unweighted counts. The descriptive denominator is 16,223 observations from 7,982 individuals; school-level and income missingness are retained in this table.

b Percentages in parentheses represent weighted estimates. Percentages use all descriptive observations in each wave, including unavailable categories; income cutpoints are defined in Section 2.3.

Supplementary Table S1 further compares the included participants with those excluded due to missing data, showing differences in observed characteristics, particularly age and school-level availability. These comparisons describe possible selection and do not establish that missingness is random.

### 3.2 Trends in physical activity time among children and adolescents

The unadjusted weighted average daily PA time among Chinese children and adolescents was 31.5, 34.3, 34.5, 15.6, and 25.8 min in 2014, 2016, 2018, 2020, and 2022, respectively. Between 2014 and 2018, average daily PA time increased by 3.0 min/day (95% CI: 0.1, 6.0); this small difference was not robust when restricting age to 10–17 or excluding values above 180 min/day. From 2020 to 2022, PA increased by 10.2 min/day (95% CI: 7.5, 12.9), with a positive difference in all tested sensitivity scenarios. The marked 2018–2020 difference spans the change in questionnaire and cannot be attributed to the pandemic. These results are presented in Table 2, with sensitivity analyses in Supplementary Table S5. The weighted percentages reporting ≥60 min/day were 16.9, 20.3, 20.2, 8.7, and 15.1%, respectively.

**Table 2:** Average daily exercise-related physical activity time among Chinese children and adolescents, 2014–2022^a^^,^^b^.

	2014	2016	2018	2020	2022
Overall^c^	31.5 (28.6, 34.3)	34.3 (31.0, 37.7)	34.5 (31.6, 37.5)	15.6 (13.7, 17.6)	25.8 (23.0, 28.7)
School Level					
Primary	26.5 (23.3, 29.7)	30.5 (26.5, 34.5)	32.1 (28.0, 36.2)	13.8 (11.4, 16.2)	23.5 (19.4, 27.6)
Middle	32.3 (28.8, 35.9)	37.4 (31.5, 43.3)	36.8 (32.3, 41.4)	15.7 (13.3, 18.1)	30.4 (25.7, 35.2)
High	41.4 (34.4, 48.3)	38.4 (32.7, 44.1)	40.2 (33.1, 47.3)	19.1 (15.2, 23.0)	25.6 (21.3, 29.9)
Gender					
Female	26.8 (22.8, 30.7)	24.1 (21.0, 27.1)	28.8 (25.0, 32.6)	12.5 (10.3, 14.6)	17.0 (14.7, 19.4)
Male	35.6 (32.1, 39.1)	45.2 (40.0, 50.5)	39.7 (35.4, 44.1)	18.6 (16.0, 21.2)	33.4 (29.1, 37.8)
Income					
Low	30.9 (24.5, 37.2)	33.3 (26.2, 40.5)	26.7 (23.4, 30.0)	11.3 (8.2, 14.4)	20.2 (15.8, 24.5)
Middle	31.9 (27.6, 36.2)	31.7 (27.0, 36.5)	34.3 (29.5, 39.1)	16.4 (13.1, 19.7)	26.2 (22.0, 30.3)
High	32.1 (28.6, 35.5)	37.2 (32.1, 42.4)	42.8 (36.9, 48.6)	18.3 (15.2, 21.3)	28.2 (23.5, 32.9)
Area					
Rural	27.8 (24.9, 30.8)	29.5 (24.9, 34.2)	30.0 (26.1, 33.9)	13.2 (11.2, 15.2)	23.0 (19.1, 26.8)
Urban	34.3 (29.9, 38.7)	36.0 (31.9, 40.1)	38.9 (35.0, 42.8)	17.5 (14.8, 20.1)	28.4 (24.5, 32.4)
Region					
North	34.7 (27.3, 42.0)	36.4 (28.8, 44.0)	37.8 (28.1, 47.5)	13.4 (9.3, 17.5)	24.8 (16.9, 32.6)
Northeast	40.1 (31.1, 49.1)	38.6 (32.5, 44.7)	41.3 (32.6, 50.1)	17.1 (12.4, 21.8)	32.7 (28.9, 36.6)
East	32.5 (25.2, 39.8)	34.1 (27.4, 40.8)	38.8 (31.6, 46.1)	16.2 (12.0, 20.3)	26.7 (20.7, 32.6)
Central	27.5 (21.9, 33.1)	30.5 (24.0, 37.1)	28.8 (24.1, 33.5)	17.1 (11.8, 22.4)	22.3 (14.9, 29.8)
South	26.0 (18.4, 33.6)	31.2 (18.4, 44.1)	32.8 (24.6, 40.9)	16.1 (10.1, 22.0)	25.0 (19.7, 30.4)
Southwest	30.2 (25.0, 35.4)	34.6 (25.7, 43.5)	30.1 (25.8, 34.4)	13.8 (9.3, 18.3)	28.1 (20.9, 35.3)
Northwest	33.9 (29.6, 38.2)	38.9 (34.2, 43.7)	39.7 (33.9, 45.4)	20.3 (16.0, 24.5)	21.0 (17.6, 24.4)

a Exercise-related physical activity time and corresponding 95% confidence intervals (CIs) are weighted estimates.

b Values are minutes/day. The descriptive sample uses available outcomes and positive weights, including observations with unavailable model covariates. School-level rows are limited to the three comparable stages.

c The overall 2018–2014 difference was 3.0 min/day (95% CI: 0.1, 6.0), and the 2022–2020 difference was 10.2 (7.5, 12.9). These are categorical-wave contrasts, not annual slopes. The 2018–2020 comparison spans a questionnaire change. Formal subgroup interaction tests are in Table 3.

### 3.3 Trends in physical activity disparities among subgroups of children and adolescents

Gender disparities persisted throughout the study, with boys consistently more active than girls; the adjusted gap peaked in 2016 at 23.0 min/day (95% CI: 17.3, 28.7), was 7.4 (4.8, 10.0) in 2020, and increased to 15.9 (10.8, 21.1) in 2022. Formal year × subgroup tests supported variation in gender differences (Holm-adjusted *p* < 0.001). Regional differences also varied in the primary analysis (Holm-adjusted *p* = 0.031), but this finding was less consistent within questionnaire periods and in sensitivity analyses; it did not establish uniform narrowing. There was limited evidence for changes in school-level, urban–rural, or income disparities (Holm-adjusted *p* = 0.251, 0.890, and 0.890, respectively). Detailed results are presented in Table 3, Supplementary Tables S3–S5, and Figure 1. Region-specific PA trend figures are provided in Supplementary Figure 1.

**Table 3:** Year × subgroup interaction tests for daily exercise-related physical activity.

Year × subgroup	Wald F	df	*p*	Holm *p*
School Level	1.78	8, 170	0.084	0.251
Gender	7.47	4, 170	<0.001	<0.001
Income	0.99	8, 170	0.445	0.890
Area	0.59	4, 170	0.670	0.890
Region	1.95	24, 170	0.008	0.031

All models include categorical survey wave, continuous age, school level, gender, household income, urban–rural residence, and region, with 1 year × subgroup interaction at a time. *n* = 14,281 observations from 7,226 individuals. Ordinary Wald F tests use 170 denominator degrees of freedom. Holm adjustment covers the five subgroup tests; individual CIs are pointwise. A regional interaction does not establish uniform narrowing.

**Figure 1:**
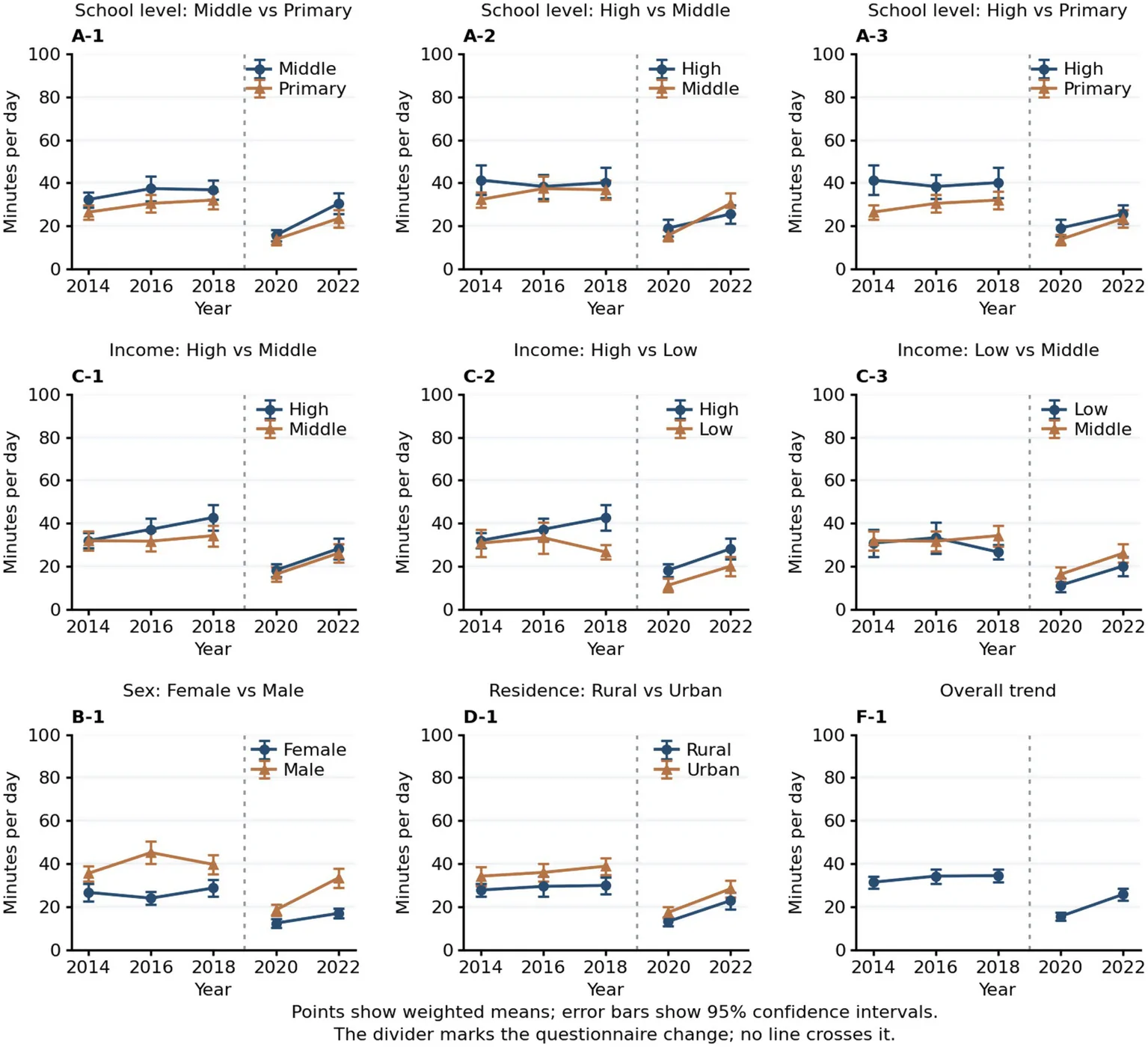
Weighted trends in exercise-related physical activity among chinese children and adolescents, 2014–2022. The nine panels retain the original school-level and income pairwise comparisons, gender, urban–rural residence, and overall series. Data are weighted using national cross-sectional weights. Error bars represent pointwise 95% confidence intervals (CIs). Lines are separated at the 2018–2020 questionnaire transition. These descriptive subgroup means are not adjusted mean differences; formal interaction tests are reported in Table 3.

### 3.4 Sociodemographic associations with the exercise-duration threshold among children and adolescents

As shown in Table 4, reporting ≥60 min/day of exercise-related PA among Chinese children and adolescents was associated with gender, household income, and urban–rural residence after mutual adjustment including age and survey year. Compared to primary schoolers, middle and high school students did not have clearly different adjusted odds (OR = 0.97, 95% CI: 0.74, 1.26; and OR = 0.93, 95% CI: 0.61, 1.40, respectively). Males had over twice the odds compared to females (OR = 2.49, 95% CI: 2.14, 2.89).

**Table 4:** Weighted logistic regression models of reporting ≥60 minutes per day of exercise-related physical activity among Chinese children and adolescents, 2014–2022^a^.

		OR (95% CI)	*p*
School level	Primary	1 [Reference]	—
	Middle	0.97 (0.74, 1.26)	0.810
	High	0.93 (0.61, 1.40)	0.720
Gender	Female	1 [Reference]	—
	Male	2.49 (2.14, 2.89)	<0.001
Income	Low	1 [Reference]	—
	Middle	1.25 (1.06, 1.49)	0.010
	High	1.44 (1.21, 1.71)	<0.001
Area	Rural	1 [Reference]	—
	Urban	1.39 (1.20, 1.62)	<0.001
Region	North	1 [Reference]	—
	Northeast	1.00 (0.74, 1.36)	0.996
	East	0.83 (0.60, 1.15)	0.272
	Central	0.69 (0.51, 0.94)	0.019
	South	0.71 (0.49, 1.03)	0.072
	Southwest	0.89 (0.64, 1.24)	0.502
	Northwest	1.07 (0.82, 1.39)	0.620
Year	2014	1 [Reference]	—
	2016	1.15 (0.96, 1.39)	0.129
	2018	1.25 (1.05, 1.49)	0.011
	2020	0.44 (0.34, 0.56)	<0.001
	2022	0.85 (0.68, 1.07)	0.176
Age	Per year	1.09 (1.02, 1.16)	0.013

a Odds ratios (ORs) are mutually adjusted for all listed variables, including categorical survey wave and continuous age; *n* = 14,281 observations from 7,226 individuals. CIs and *p*-values use the survey-design approximation described in Section 2.4. The outcome is an exploratory exercise-duration threshold, not WHO guideline adherence. The 2022 vs. 2020 OR is 1.94 (95% CI: 1.50, 2.51).

Children and adolescents from middle- and high-income households had higher odds of reporting ≥60 min/day of exercise-related PA than those from low-income families (OR = 1.25, 95% CI: 1.06, 1.49; and OR = 1.44, 95% CI: 1.21, 1.71, respectively). Urban children and adolescents also had higher odds compared to their rural counterparts (OR = 1.39, 95% CI: 1.20, 1.62). In contrast, those living in the central region had lower odds than those in the northern region (OR = 0.69, 95% CI: 0.51, 0.94); the CIs for the other regional contrasts included 1. The odds were higher in 2022 than in 2020 (OR = 1.94, 95% CI: 1.50, 2.51), within the same questionnaire period.

## 4 Discussion

Using nationally representative data from the CFPS spanning 2014 to 2022, this study investigated temporal patterns in exercise-related PA among Chinese children and adolescents, as well as associations between sociodemographic factors and exercise participation. The unadjusted weighted mean difference in daily exercise-related PA time was 3.05 min/day (95% CI, 0.07–6.02) for 2018 versus 2014 and 10.20 min/day (7.48–12.92) for 2022 versus 2020. Notably, males consistently reported higher levels of exercise-related PA than females; differences by sex and region varied across waves in the primary analysis, whereas formal interactions did not support changing differences by school level, urban–rural residence or household income. Regional evidence was less consistent in sensitivity analyses. Gender, household income and urban–rural residence were associated with reporting at least 60 min/day of exercise-related PA, an exploratory duration threshold rather than WHO guideline adherence.

### 4.1 Trends in exercise-related physical activity over time

Previous research has shown that insufficient PA remained common globally, although the 2001–2016 trend differed between boys and girls (1). In the present study, between 2014 and 2018—prior to the COVID-19 pandemic—average daily exercise-related PA time among Chinese children and adolescents increased modestly. However, this difference became uncertain when analyses were restricted to ages 10–17 or excluded durations above 180 min/day. A Chinese school survey reported declines in activity classification for several groups from 2017 to 2019 (9); differences in populations and measurement preclude interpreting these findings as directly contradictory.

The Chinese government placed a strong policy emphasis on promoting PA among children and adolescents during this period. For instance, in 2014, China introduced the revised National Student Physical Fitness Standards, mandating annual physical fitness assessments from primary through university levels, with results incorporated into school performance evaluations (23). In 2016, the State Council issued the Opinions on Strengthening School Physical Education to Promote the Physical and Mental Health of Students, which emphasized increasing extracurricular PA opportunities (24). Whether these policies and subsequent implementation efforts contributed to increased PA opportunities and engagement among children and adolescents cannot be determined from the present analysis. Future evaluations should assess implementation quality through delivered PE time, teacher availability and facility access, and link these measures to activity outcomes.

However, exercise-related PA was lower in 2020 than in 2018 and higher in 2022 than in 2020; these observations do not indicate a continuous later-period decline. The 2018–2020 difference spanned the onset of the COVID-19 era, when studies reported reduced PA during periods of disrupted schooling and restricted mobility (3). At the same time, the CFPS PA items were revised in 2020 (including changes in reference period and response format), which may affect comparability around the 2018–2020 transition. Therefore, the magnitude of the apparent drop between 2018 and 2020 should be interpreted cautiously and may reflect both real-world change and methodological differences. Nevertheless, these wave differences highlight the need for effective strategies to protect and promote PA among children and adolescents during public health crises and other exceptional circumstances. The analysis was not designed to assess post-pandemic recovery.

International comparisons suggest that appropriate strategies should reflect local opportunities for PA. For example, in Global Matrix 4.0, the four countries with low or medium human development had stronger grades for active play and transport than for community environments (25). This small and heterogeneous group does not represent all low- and middle-income countries, and national development categories are not equivalent to household income. These findings underscore the value of tailored, context-specific policies and interventions to sustain and promote PA among youth, and the importance of distinguishing exercise from other activity domains.

### 4.2 Disparities across demographic subgroups

Beyond these crisis-related implications, the results of this study indicate disparities in exercise-related PA across gender, household income, urban–rural residence and geographic region. Specifically, formal wave-by-subgroup interactions supported variation in sex and regional differences, but not changing differences by school level, urban–rural residence or household income. These patterns should not be interpreted as evidence that regional gaps uniformly narrowed or that income disparities widened. Possible roles of national policy initiatives, economic development and sociocultural changes cannot be determined from these associations.

In 2012, the Chinese government issued the Guidelines on Further Strengthening School Physical Education (26). The Ministry of Education’s 2021 guidance specifies four weekly PE classes in primary grades 1–2, three in primary grades 3–6 and lower-secondary school, and two in upper-secondary school (27). Implementation should be evaluated through delivered lesson time and teacher coverage; the present data do not establish that these policies narrowed school-stage differences in exercise-related PA. Additionally, the National Development Plan for Children in Poor Areas, introduced in 2014 (28), sought to improve access to facilities and equipment in disadvantaged areas. The plan explicitly called for improvements in school-based physical infrastructure and resource allocation in economically disadvantaged rural regions, whose implementation could be monitored through facility availability, equipment access and participation by urban–rural residence.

Furthermore, regional differences in exercise-related PA varied across waves (Holm-adjusted *p* = 0.031), but the interaction did not establish uniform narrowing and was less consistent in sensitivity analyses. The data were not linked to local weather, facility access or policy implementation, so these explanations for regional variation could not be tested.

With regard to household income, the findings of this study indicate differences in exercise-related PA time among children and adolescents from different income groups. The adjusted high-versus-low-income difference was 13.19 min/day in 2018 (95% CI, 5.95–20.43) and 8.67 in 2022 (2.34–15.00), but the wave-by-income interaction did not support changing differences (Holm-adjusted *p* = 0.890). These estimates do not support a continuously widening gap. Several possible explanations may account for these income-related differences. First, program fees and travel costs may limit PA opportunities for low-income households. Second, middle- and high-income families may have more financial resources to invest in sports clubs, private training, or extracurricular programs for their children (8). Additionally, unequal access to safe spaces, equipment and caregivers’ time could contribute to differences in PA opportunities. However, these explanations remain speculative and warrant further investigation. Future research should explore the specific mechanisms underlying PA differences across income groups to inform targeted interventions. Policies could combine universal school PA opportunities with free or subsidized programs, equipment loans and safe spaces for groups with lower participation, including girls and families facing financial barriers, while monitoring uptake and continued participation.

### 4.3 Sociodemographic associations with an exercise duration threshold

Previous studies have shown that PA levels are generally higher among younger students (4), males (1), children from high-income families (8), and urban residents (6), compared to their counterparts in higher school grades, females, low-income families, and rural areas. The findings of the present study are largely consistent with these sex, income and residence patterns for the exploratory exercise-duration threshold. However, after age adjustment, lower-secondary and upper-secondary school respondents did not have clearly higher odds of reporting at least 60 min/day than primary-school respondents (OR = 0.97; 95% CI, 0.74–1.26, and OR = 0.93; 0.61–1.40, respectively). School stage should therefore be distinguished from chronological age when interpreting this threshold. Comparisons with previous studies also depend on the way PA was assessed in the CFPS. The CFPS questionnaires primarily focus on exercise/fitness-related PA, rather than comprehensively covering unstructured activity. For example, the 2020–2022 question describes indoor or outdoor PA for the purpose of strengthening the body or promoting mental well-being, excluding cycling or walking solely to travel to or from work; examples in the earlier questionnaires include walking. These domain differences should not be used to explain an independent school-stage association that was not supported after age adjustment. The exercise-duration threshold does not establish WHO guideline adherence because intensity and muscle- and bone-strengthening activity are not consistently measured (2).

### 4.4 Strengths and limitations

A major strength of this study lies in the use of multiple waves of nationally representative data from the CFPS spanning 2014–2022, as well as the formal analysis of how exercise-related PA disparities across demographic subgroups vary over time.

Despite these strengths, several limitations should be acknowledged. First, the CFPS modified the PA items in 2020, including changes in the reference period (past week vs. past 12 months) and response format (continuous duration vs. categorical frequency combined with typical session duration). Weekly exercise-related PA minutes for 2020–2022 therefore required conversion from frequency categories, which involves approximation. No calibration subsample answered both questionnaire versions over the same reference period. These measurement differences may have introduced non-equivalence and systematic error when comparing PA estimates between the 2014–2018 and 2020–2022 periods. Accordingly, the marked difference observed between 2018 and 2020 may partly reflect methodological change in addition to real-world changes (including potential pandemic-related effects), and caution is needed when interpreting cross-period differences. Second, the PA items primarily capture exercise/fitness-related activities (including PE classes for students) and may be more salient for structured or planned exercise. As a result, respondents may underreport unstructured or incidental activity (e.g., free play or informal peer interaction), particularly common among younger children. In addition, the 2020–2022 item explicitly excludes walking/cycling performed solely to travel to or from work, whereas earlier examples include walking; our measure does not comprehensively capture total PA or establish MVPA intensity. Third, direct validation of these items against a comparable activity measure was unavailable; previous use in peer-reviewed studies does not establish psychometric validity or equivalence across waves. Fourth, we found differences in demographic characteristics between participants included in the final analysis and those excluded due to missing data, suggesting that selection bias cannot be ruled out and potentially limiting generalizability. Descriptive estimates retain otherwise eligible observations with missing covariates, while the adjusted models require complete covariates; weighting does not eliminate the resulting selection concerns. Finally, the available data did not include in-depth qualitative or implementation information to explore contextual drivers of the observed differences, which warrants further qualitative or mixed-methods research directly engaging youth populations. Because CFPS follows a panel, individuals can contribute repeated observations and age into or out of the eligible age range. Age adjustment and stable PSU clustering do not separate age, period and birth-cohort effects. The variance estimator uses available baseline PSU and sampling-frame information without reconstructing every implicit stratum or finite population correction.

## 5 Conclusion

This study found a modest difference in exercise-related PA among Chinese children and adolescents between 2014 and 2018, and higher reported duration in 2022 than in 2020. The 2018–2020 comparison was limited by the questionnaire change, and these data do not establish causal policy or pandemic effects. In the primary analysis, sex and regional differences varied across waves, whereas formal interactions did not support changing differences by school level, urban–rural residence or household income. The regional findings were less consistent in sensitivity analyses. From both public health and educational perspectives, these findings underscore the importance of implementing subgroup-specific PA promotion strategies. Future policies and research should closely monitor PA differences among children and adolescents from different income backgrounds and develop targeted interventions to reduce these disparities and promote PA equity.

## Data Availability

The original contributions presented in the study are included in the article/Supplementary material, further inquiries can be directed to the corresponding author.
