# Supplementary material for: Evolving disparities in exercise-related physical activity among Chinese children and adolescents: evidence from a national repeated cross-sectional analysis, 2014–2022
Source: Front Public Health. 2026 Oct 14;14:1883019. doi: 10.3389/fpubh.2026.1883019 (PMC13713619; doi:10.3389/fpubh.2026.1883019)
Supplement: Supplementary file 1 [file Data_Sheet_1.docx]

Supplementary materials

These materials document sample selection, measurement and weighting, detailed subgroup estimates, and sensitivity analyses. All estimates describe exercise-related activity; the 60-minute threshold does not measure WHO guideline adherence.

# Supplementary Table S1 Sample flow and missing data

S1a Sequential sample flow

| Record count | 2014 | 2016 | 2018 | 2020 | 2022 |
| --- | --- | --- | --- | --- | --- |
| Age eligible records | 4,380 | 3,923 | 3,782 | 3,036 | 2,985 |
| Exercise outcome unavailable | 596 | 274 | 259 | 231 | 125 |
| Valid exercise outcome | 3,784 | 3,649 | 3,523 | 2,805 | 2,860 |
| Weight unavailable after valid outcome | 54 | 80 | 79 | 102 | 83 |
| Weighted descriptive sample | 3,730 | 3,569 | 3,444 | 2,703 | 2,777 |
| Complete covariate model sample | 3,167 | 3,207 | 3,090 | 2,346 | 2,471 |
| Explicit zero exercise in descriptive sample | 1,104 | 1,312 | 1,113 | 1,067 | 673 |

Counts are person-wave observations. The descriptive sample has 7,982 unique people; the model sample has 7,226. Of 18,106 age-eligible records, 1,485 lack a valid outcome and 398 more lack a positive weight. A further 1,942 lack the required model covariates. Skipped duration following an explicit no-exercise response is zero, not missing.

S1b Covariate availability in the descriptive sample

| Unavailable covariate | 2014 | 2016 | 2018 | 2020 | 2022 |
| --- | --- | --- | --- | --- | --- |
| School outside selected stages or unavailable | 408 | 278 | 238 | 131 | 118 |
| Residence unavailable | 58 | 27 | 25 | 68 | 11 |
| Region unavailable | 0 | 0 | 0 | 0 | 0 |
| Income unavailable | 129 | 65 | 113 | 196 | 188 |
| Sex unavailable | 0 | 0 | 0 | 0 | 0 |

These counts overlap and must not be summed as sequential exclusions. Income and school-stage availability are handled separately from the activity outcome. No missing-income category was imputed to the middle group.

S1c Included and excluded record profiles

| Wave and sample | n | Mean age | Female % | Urban % | School unavailable n | Income unavailable n |
| --- | --- | --- | --- | --- | --- | --- |
| 2014 Descriptive included | 3730 | 13.9 | 47.9 | 41.8 | 408 | 129 |
| 2014 Descriptive excluded | 650 | 14.1 | 49.1 | 29.2 | 540 | 52 |
| 2014 Model included | 3167 | 13.6 | 48.5 | 43.3 | 0 | 0 |
| 2014 Descriptive included but model excluded | 563 | 16.1 | 44.4 | 33.9 | 408 | 129 |
| 2016 Descriptive included | 3569 | 13.8 | 46.6 | 42.3 | 278 | 65 |
| 2016 Descriptive excluded | 354 | 15.2 | 50.0 | 33.9 | 218 | 29 |
| 2016 Model included | 3207 | 13.6 | 46.8 | 43.2 | 0 | 0 |
| 2016 Descriptive included but model excluded | 362 | 15.9 | 44.2 | 34.0 | 278 | 65 |
| 2018 Descriptive included | 3444 | 13.8 | 46.9 | 42.0 | 238 | 113 |
| 2018 Descriptive excluded | 338 | 14.4 | 55.9 | 21.3 | 70 | 49 |
| 2018 Model included | 3090 | 13.6 | 46.9 | 42.1 | 0 | 0 |
| 2018 Descriptive included but model excluded | 354 | 15.8 | 46.9 | 41.5 | 238 | 113 |
| 2020 Descriptive included | 2703 | 13.8 | 47.7 | 41.7 | 131 | 196 |
| 2020 Descriptive excluded | 333 | 13.8 | 45.6 | 32.1 | 42 | 46 |
| 2020 Model included | 2346 | 13.6 | 47.1 | 43.1 | 0 | 0 |
| 2020 Descriptive included but model excluded | 357 | 15.0 | 51.3 | 32.2 | 131 | 196 |
| 2022 Descriptive included | 2777 | 13.8 | 46.4 | 44.1 | 118 | 188 |
| 2022 Descriptive excluded | 208 | 14.0 | 51.9 | 42.8 | 24 | 52 |
| 2022 Model included | 2471 | 13.6 | 46.5 | 44.8 | 0 | 0 |
| 2022 Descriptive included but model excluded | 306 | 15.1 | 46.1 | 38.9 | 118 | 188 |

All values in S1c are unweighted. Percentages use all records in the corresponding row as denominator; an unavailable category is not included in the numerator. Excluded records differ in observed composition, particularly age and school-stage availability. These descriptive comparisons do not establish a missing-at-random mechanism. Missing values were not imputed.

# Supplementary Table S2 Measurement and weighting

S2a Exercise variables and outcome derivation

| Wave and source | Variables | Operational rule |
| --- | --- | --- |
| 2014 and 2016 child self-response | wl3a and wl3b | Past-week exercise frequency and total hours; explicit frequency 0 → 0 min/day; otherwise hours × 60 / 7. |
| 2014 and 2016 adult self-response | qp701 and qp702 | Same past-week total-duration approach. Only age-eligible respondents are included. |
| 2018 person self-response | qp701 and qp702 | Past-week total hours × 60 / 7; explicit no exercise is zero. |
| 2020 and 2022 person self-response | qp701n and qp702n | Usual frequency and typical minutes per occasion in past 12 months; category score × minutes / 7; code 8 never → 0. |

The child self-response module uses age-appropriate self-report. A skipped duration code (−8) after explicit no exercise is structurally determined; other negative response codes are treated as missing. There were no cases with missing frequency but an observed nonnegative total duration in the early waves. All 5,438 explicit no-exercise records among age-eligible respondents had structurally skipped duration; 5,269 had a positive analysis weight.

S2b Later frequency scoring in sessions per week

| Code | Response category | Main | Lower scenario | Higher scenario |
| --- | --- | --- | --- | --- |
| 1 | Less than once per month | 0.125 | 0.125 | 0.25 |
| 2 | At least monthly but less than weekly | 0.5 | 0.25 | 0.75 |
| 3 | 1–2 times per week | 1.5 | 1 | 2 |
| 4 | 3–4 times per week | 3.5 | 3 | 4 |
| 5 | At least 5 times per week | 5.5 | 5 | 6 |
| 6 | Once per day | 7 | 7 | 7 |
| 7 | At least twice per day | 14 | 14 | 21 |
| 8 | Never | 0 | 0 | 0 |

Scenarios are alternative representative scores, not statistical identification bounds. The original mapping sensitivity changes only category 1 from 0.125 to 0.25 sessions/week relative to the main mapping. The uppermost category is open-ended, so true frequency cannot be recovered exactly.

S2c National cross-sectional weight variables

| Source file | Original national weight variable | Main analysis |
| --- | --- | --- |
| cfps2014child_201906.dta | rswt_natcs14 | Released national cross-sectional weight |
| cfps2014adult_201906.dta | rswt_natcs14 | Released national cross-sectional weight |
| cfps2016child_201906.dta | rswt_natcs16 | Released national cross-sectional weight |
| cfps2016adult_201906.dta | rswt_natcs16 | Released national cross-sectional weight |
| cfps2018person_202012.dta | rswt_natcs18n | Released national cross-sectional weight |
| cfps2020person_202306.dta | rswt_natcs20n | 2020 main uses rswtps_natcs20n |
| cfps2022person_202410.dta | rswt_natcs22n | Released national cross-sectional weight |

For 2020, rswtps_natcs20n is the recommended post-stratified national cross-sectional weight; rswt_natcs20n is used only in the alternative-weight analysis. Weight releases differ in construction and scale. The manuscript does not claim that all five releases use an identical post-stratification procedure. Baseline PSU and six sampling-frame groups are linked from the cross-year identification file. The 176-PSU grid contains 32, 16, 16, 16, 16, and 80 PSUs in the six respective groups. Each pooled wave receives the same total weight before outcome/covariate exclusions.

S2d Income cutpoints in Chinese yuan

| Wave | 30th percentile | 70th percentile | Observed income records |
| --- | --- | --- | --- |
| 2014 | 25,000 | 50,000 | 4,199 |
| 2016 | 25,000 | 60,000 | 3,829 |
| 2018 | 30,000 | 68,600 | 3,620 |
| 2020 | 35,000 | 80,000 | 2,794 |
| 2022 | 45,000 | 100,000 | 2,745 |

Cutpoints are unweighted among all age-eligible youth records with observed annual total household income. Special negative codes are excluded before calculating cutpoints. Low ≤30th-percentile value; high ≥70th-percentile value; middle between them. These categories do not equivalize household size or identify absolute poverty. Province mapping is supplied in the code.

# Supplementary Table S3 Subgroup and age specific mean duration

S3a Sociodemographic subgroup means in minutes per day

| Group | 2014 | 2016 | 2018 | 2020 | 2022 |
| --- | --- | --- | --- | --- | --- |
| Sex: Female | 26.8 (22.8, 30.7) n = 1,786 | 24.1 (21.0, 27.1) n = 1,662 | 28.8 (25.0, 32.6) n = 1,616 | 12.5 (10.3, 14.6) n = 1,288 | 17.0 (14.7, 19.4) n = 1,289 |
| Sex: Male | 35.6 (32.1, 39.1) n = 1,944 | 45.2 (40.0, 50.5) n = 1,907 | 39.7 (35.4, 44.1) n = 1,828 | 18.6 (16.0, 21.2) n = 1,415 | 33.4 (29.1, 37.8) n = 1,488 |
| Residence: Rural | 27.8 (24.9, 30.8) n = 2,111 | 29.5 (24.9, 34.2) n = 2,034 | 30.0 (26.1, 33.9) n = 1,972 | 13.2 (11.2, 15.2) n = 1,508 | 23.0 (19.1, 26.8) n = 1,540 |
| Residence: Urban | 34.3 (29.9, 38.7) n = 1,561 | 36.0 (31.9, 40.1) n = 1,508 | 38.9 (35.0, 42.8) n = 1,447 | 17.5 (14.8, 20.1) n = 1,127 | 28.4 (24.5, 32.4) n = 1,226 |
| School stage: Primary | 26.5 (23.3, 29.7) n = 1,376 | 30.5 (26.5, 34.5) n = 1,461 | 32.1 (28.0, 36.2) n = 1,408 | 13.8 (11.4, 16.2) n = 1,081 | 23.5 (19.4, 27.6) n = 1,120 |
| School stage: Lower secondary | 32.3 (28.8, 35.9) n = 1,233 | 37.4 (31.5, 43.3) n = 1,146 | 36.8 (32.3, 41.4) n = 1,134 | 15.7 (13.3, 18.1) n = 931 | 30.4 (25.7, 35.2) n = 847 |
| School stage: Upper secondary | 41.4 (34.4, 48.3) n = 713 | 38.4 (32.7, 44.1) n = 684 | 40.2 (33.1, 47.3) n = 664 | 19.1 (15.2, 23.0) n = 560 | 25.6 (21.3, 29.9) n = 692 |
| Household income: Low | 30.9 (24.5, 37.2) n = 1,100 | 33.3 (26.2, 40.5) n = 1,102 | 26.7 (23.4, 30.0) n = 1,183 | 11.3 (8.2, 14.4) n = 767 | 20.2 (15.8, 24.5) n = 775 |
| Household income: Middle | 31.9 (27.6, 36.2) n = 1,131 | 31.7 (27.0, 36.5) n = 1,328 | 34.3 (29.5, 39.1) n = 1,153 | 16.4 (13.1, 19.7) n = 937 | 26.2 (22.0, 30.3) n = 912 |
| Household income: High | 32.1 (28.6, 35.5) n = 1,370 | 37.2 (32.1, 42.4) n = 1,074 | 42.8 (36.9, 48.6) n = 995 | 18.3 (15.2, 21.3) n = 803 | 28.2 (23.5, 32.9) n = 902 |

Cells show the weighted mean, pointwise 95% CI, and unweighted n. Each subgroup uses available activity, positive weights, and an observed category for that dimension; it is not restricted to the complete-covariate model sample.

S3b Age band means in minutes per day

| Group | 2014 | 2016 | 2018 | 2020 | 2022 |
| --- | --- | --- | --- | --- | --- |
| Age 10-12 | 27.6 (24.2, 30.9) n = 1,272 | 30.8 (26.8, 34.8) n = 1,321 | 32.3 (27.9, 36.7) n = 1,225 | 14.0 (11.4, 16.5) n = 976 | 23.1 (19.0, 27.2) n = 1,019 |
| Age 13-15 | 30.8 (27.1, 34.6) n = 1,261 | 36.5 (30.6, 42.4) n = 1,116 | 35.2 (31.2, 39.3) n = 1,186 | 15.4 (12.7, 18.0) n = 922 | 29.5 (24.4, 34.6) n = 898 |
| Age 16-18 | 34.2 (29.4, 38.9) n = 1,197 | 35.0 (30.5, 39.5) n = 1,132 | 36.2 (30.8, 41.5) n = 1,033 | 18.1 (14.3, 21.8) n = 805 | 25.3 (21.4, 29.2) n = 860 |

Age bands are descriptive intervals; they are not biological categories of childhood and adolescence. Adjusted models use continuous age.

S3c Regional means in minutes per day

| Group | 2014 | 2016 | 2018 | 2020 | 2022 |
| --- | --- | --- | --- | --- | --- |
| Region: North | 34.7 (27.3, 42.0) n = 399 | 36.4 (28.8, 44.0) n = 392 | 37.8 (28.1, 47.5) n = 377 | 13.4 (9.3, 17.5) n = 338 | 24.8 (16.9, 32.6) n = 391 |
| Region: Northeast | 40.1 (31.1, 49.1) n = 393 | 38.6 (32.5, 44.7) n = 349 | 41.3 (32.6, 50.1) n = 295 | 17.1 (12.4, 21.8) n = 229 | 32.7 (28.9, 36.6) n = 198 |
| Region: East | 32.5 (25.2, 39.8) n = 540 | 34.1 (27.4, 40.8) n = 528 | 38.8 (31.6, 46.1) n = 526 | 16.2 (12.0, 20.3) n = 422 | 26.7 (20.7, 32.6) n = 436 |
| Region: Central | 27.5 (21.9, 33.1) n = 680 | 30.5 (24.0, 37.1) n = 669 | 28.8 (24.1, 33.5) n = 637 | 17.1 (11.8, 22.4) n = 486 | 22.3 (14.9, 29.8) n = 563 |
| Region: South | 26.0 (18.4, 33.6) n = 550 | 31.2 (18.4, 44.1) n = 437 | 32.8 (24.6, 40.9) n = 446 | 16.1 (10.1, 22.0) n = 378 | 25.0 (19.7, 30.4) n = 350 |
| Region: Southwest | 30.2 (25.0, 35.4) n = 550 | 34.6 (25.7, 43.5) n = 577 | 30.1 (25.8, 34.4) n = 568 | 13.8 (9.3, 18.3) n = 395 | 28.1 (20.9, 35.3) n = 379 |
| Region: Northwest | 33.9 (29.6, 38.2) n = 618 | 38.9 (34.2, 43.7) n = 617 | 39.7 (33.9, 45.4) n = 595 | 20.3 (16.0, 24.5) n = 455 | 21.0 (17.6, 24.4) n = 460 |

# Supplementary Table S4 Adjusted subgroup differences by wave

| Contrast | 2014 | 2016 | 2018 | 2020 | 2022 |
| --- | --- | --- | --- | --- | --- |
| Male versus Female | 9.5 (4.5, 14.5) | 23.0 (17.3, 28.7) | 14.0 (7.9, 20.1) | 7.4 (4.8, 10.0) | 15.9 (10.8, 21.1) |
| Urban versus Rural | 4.1 (-1.4, 9.6) | 5.3 (-0.4, 11.0) | 6.7 (1.2, 12.3) | 2.6 (-0.6, 5.8) | 4.5 (-0.6, 9.5) |
| Lower secondary versus Primary | 2.2 (-3.4, 7.8) | 2.9 (-3.1, 9.0) | 0.4 (-6.5, 7.3) | -1.5 (-6.1, 3.1) | 5.3 (-1.0, 11.7) |
| Upper secondary versus Primary | 8.5 (-0.7, 17.6) | 1.4 (-6.8, 9.6) | 0.7 (-10.2, 11.5) | -0.1 (-7.5, 7.2) | -2.2 (-9.7, 5.3) |
| Northeast versus North | 0.7 (-9.1, 10.6) | 4.7 (-5.4, 14.7) | 2.6 (-12.3, 17.4) | 0.7 (-5.4, 6.8) | 7.4 (-0.3, 15.1) |
| East versus North | -3.1 (-15.1, 8.9) | -2.8 (-13.5, 7.9) | -2.5 (-15.4, 10.4) | 0.3 (-5.6, 6.1) | 1.1 (-7.7, 9.9) |
| Central versus North | -8.1 (-17.7, 1.6) | -5.4 (-15.4, 4.6) | -10.9 (-22.1, 0.2) | -0.4 (-5.6, 4.9) | -1.9 (-11.8, 8.0) |
| South versus North | -12.4 (-23.6, -1.2) | -4.0 (-19.8, 11.8) | -5.5 (-18.4, 7.4) | 3.3 (-3.4, 10.0) | 1.5 (-7.2, 10.2) |
| Southwest versus North | -3.0 (-13.0, 7.1) | 1.0 (-11.2, 13.2) | -7.6 (-19.1, 3.9) | 0.6 (-5.8, 7.0) | 3.2 (-7.0, 13.5) |
| Northwest versus North | -0.2 (-9.5, 9.2) | 5.7 (-2.9, 14.2) | 0.7 (-11.6, 12.9) | 8.2 (2.1, 14.4) | -0.7 (-8.0, 6.7) |
| Middle versus Low | -0.0 (-7.7, 7.6) | -0.2 (-8.6, 8.2) | 5.1 (-0.8, 11.0) | 3.8 (-0.3, 8.0) | 3.9 (-2.1, 9.8) |
| High versus Low | 0.8 (-7.3, 8.9) | 2.5 (-6.6, 11.6) | 13.2 (5.9, 20.4) | 7.5 (3.2, 11.8) | 8.7 (2.3, 15.0) |

Cells show the adjusted mean difference (minutes/day) and pointwise 95% CI. Each dimension is estimated from its own wave-by-subgroup linear model with categorical wave, continuous age, sex, residence, school stage, income group, and region. All models use 14,281 complete observations. These estimates are not the differences between the unadjusted available-case means in Table S3. The five omnibus temporal-heterogeneity tests, with Holm adjustment, are reported in main Table 3.

# Supplementary Table S5 Wave contrasts and sensitivity analyses

S5a Wave contrasts in mean exercise duration

| Contrast | Mean difference (95% CI) | P | Same questionnaire period |
| --- | --- | --- | --- |
| 2016 minus 2014 | 2.8 (-0.6 to 6.3) | 0.107 | Yes |
| 2018 minus 2014 | 3.0 (0.1 to 6.0) | 0.045 | Yes |
| 2018 minus 2016 | 0.2 (-3.4 to 3.8) | 0.907 | Yes |
| 2020 minus 2018 | -18.9 (-21.7 to -16.2) | <0.001 | No |
| 2022 minus 2020 | 10.2 (7.5 to 12.9) | <0.001 | Yes |
| 2022 minus 2014 | -5.7 (-9.3 to -2.0) | 0.003 | No |

Unadjusted weighted ratio contrasts use the common stable PSU grid, retaining covariance across waves. Cross-period contrasts are descriptive and cannot isolate true behavioral change. These P values are unadjusted; they are not the Holm-adjusted interaction tests.

S5b Covariate adjusted contrasts within questionnaire periods

| Outcome | Contrast | Estimate (95% CI) | P |
| --- | --- | --- | --- |
| PA_Minutes | 2018 versus 2014 | 3.67 (0.34 to 6.99) | 0.031 |
| PA_Minutes | 2022 versus 2020 | 9.97 (6.97 to 12.96) | <0.001 |
| Benchmark | 2018 versus 2014 | 1.25 (1.05 to 1.49) | 0.011 |
| Benchmark | 2022 versus 2020 | 1.94 (1.50 to 2.51) | <0.001 |

PA_Minutes estimates are mean differences in min/day; Benchmark estimates are odds ratios for ≥60 min/day of exercise. Both models include categorical wave, continuous age, sex, residence, school stage, income, and region.

S5c Sensitivity of within questionnaire duration contrasts

| Scenario | 2018 minus 2014 (95% CI) | 2022 minus 2020 (95% CI) | n in 2020 / 2022 |
| --- | --- | --- | --- |
| Ages 10–17 | 3.1 (-0.4 to 6.6) | 11.1 (8.0 to 14.2) | 2,438 / 2,468 |
| Exclude >180 min/day | 1.2 (-1.1 to 3.5) | 6.6 (4.4 to 8.8) | 2,694 / 2,739 |
| Lower category scores | 3.0 (0.1 to 6.0) | 10.1 (7.4 to 12.8) | 2,703 / 2,777 |
| Higher category scores | 3.0 (0.1 to 6.0) | 13.9 (10.6 to 17.3) | 2,703 / 2,777 |
| Original frequency mapping | 3.0 (0.1 to 6.0) | 10.2 (7.5 to 12.9) | 2,703 / 2,777 |
| 2020 nonresponse-only weight | 3.0 (0.1 to 6.0) | 11.1 (8.4 to 13.8) | 2,703 / 2,777 |

Estimates are differences in weighted min/day. The primary 2018–2014 contrast is 3.0 (0.1 to 6.0); the primary 2022–2020 contrast is 10.2 (7.5 to 12.9). The >180-min/day exclusion removes 57, 61, 74, 9, and 38 observations by wave (239 in total) and is an influence diagnostic, not a validated validity threshold.

S5d Temporal heterogeneity under alternative specifications

| Scenario | Sex | Residence | School stage | Region | Income |
| --- | --- | --- | --- | --- | --- |
| Ages 10–17 | <0.001 | 0.705 | 0.198 | 0.051 | 0.705 |
| Exclude >180 min/day | 0.002 | 0.023 | 0.052 | 0.010 | 0.789 |
| Lower category scores | <0.001 | 0.953 | 0.174 | 0.025 | 0.953 |
| Higher category scores | <0.001 | 0.856 | 0.352 | 0.059 | 0.856 |
| Original frequency mapping | <0.001 | 0.893 | 0.248 | 0.032 | 0.893 |
| 2020 nonresponse-only weight | <0.001 | 0.845 | 0.151 | 0.039 | 0.845 |
| No age adjustment | <0.001 | 0.896 | 0.271 | 0.033 | 0.896 |
| Additional mode adjustment | <0.001 | 0.895 | 0.230 | 0.020 | 0.895 |
| 2014–2018 only | 0.007 | 1.000 | 1.000 | 1.000 | 0.792 |
| 2020–2022 only | 0.022 | 1.000 | 0.079 | 0.147 | 1.000 |

Entries are Holm-adjusted P values, adjusted over five subgroup tests within each scenario. Sensitivity scenarios themselves are not adjusted as one larger multiplicity family; they are exploratory. The full estimates and raw P values are in the aggregate results.

S5e Selected odds ratios under alternative specifications

| Contrast | Main | Ages 10–17 | No age adjustment | Additional mode adjustment |
| --- | --- | --- | --- | --- |
| Male versus female | 2.49 (2.14 to 2.89) | 2.48 (2.10 to 2.92) | 2.49 (2.15 to 2.90) | 2.48 (2.14 to 2.88) |
| Urban versus rural | 1.39 (1.20 to 1.62) | 1.39 (1.20 to 1.62) | 1.38 (1.19 to 1.60) | 1.39 (1.20 to 1.61) |
| Lower secondary versus primary | 0.97 (0.74 to 1.26) | 1.08 (0.81 to 1.42) | 1.25 (1.07 to 1.47) | 0.96 (0.74 to 1.25) |
| Upper secondary versus primary | 0.93 (0.61 to 1.40) | 0.99 (0.64 to 1.52) | 1.48 (1.26 to 1.74) | 0.91 (0.61 to 1.36) |
| Middle versus low income | 1.25 (1.06 to 1.49) | 1.37 (1.14 to 1.65) | 1.24 (1.05 to 1.48) | 1.26 (1.06 to 1.49) |
| High versus low income | 1.44 (1.21 to 1.71) | 1.55 (1.30 to 1.85) | 1.42 (1.19 to 1.69) | 1.44 (1.21 to 1.70) |

All values are ORs (95% CI) for the exploratory exercise-duration threshold. Models otherwise contain the same main effects. No-age estimates illustrate why school stage should not be interpreted independently of age. Additional personal interview-mode adjustment does not identify the questionnaire-change effect.

S5f Variance calculation and verification

For a weighted mean μ = Σwy/Σw, an observation contributes w(y−μ)/Σw to its PSU total. The variance is the sum across sampling-frame groups h of mh/(mh−1) times the sum of squared deviations of PSU totals from their within-group mean. For regression, analogous weighted score totals form the sandwich covariance with the model derivative matrix. Domain analyses retain zero-contribution PSUs. Pooled scores use a common PSU identity across waves, retaining cross-wave covariance. Intervals use t170; joint interactions use ordinary F(q,170). No Rao–Scott or finite-population adjustment is claimed.

Independent implementations matched all five wave means and standard errors, three regional-domain estimates, and the 2022–2020 contrast to numerical tolerance below 10^−10. This verifies the implemented variance calculations against an independent calculation, not against a fully reconstructed confidential CFPS sampling design. Source-variable mappings and verification notes are supplied with the accompanying code.

# Supplementary Figure S1 Regional exercise duration

Regional weighted means and pointwise design-aware 95% confidence intervals; subgroup sample sizes and numerical estimates appear in Table S3c. Lines connect only waves sharing the questionnaire format. Overall regional heterogeneity across 2014–2022 does not imply uniform convergence, and is less consistent in within-instrument analyses.
